# Redox Regulation of Autophagy in Cancer: Mechanism, Prevention and Therapy

**DOI:** 10.3390/life13010098

**Published:** 2022-12-29

**Authors:** Jingqiu He, Lixia Dong, Li Luo, Kui Wang

**Affiliations:** 1 West China School of Basic Medical Sciences and Forensic Medicine, Sichuan University, Chengdu 610041, China; 2 Center for Reproductive Medicine, Department of Gynecology and Obstetrics, West China Second University Hospital, Sichuan University, Chengdu 610041, China; 3 Key Laboratory of Birth Defects and Related Diseases of Women and Children (Sichuan University), Ministry of Education, Chengdu 610041, China

**Keywords:** redox regulation, ROS, autophagy, redox modification, cancer therapy

## Abstract

Reactive oxygen species (ROS), products of normal cellular metabolism, play an important role in signal transduction. Autophagy is an intracellular degradation process in response to various stress conditions, such as nutritional deprivation, organelle damage and accumulation of abnormal proteins. ROS and autophagy both exhibit double-edged sword roles in the occurrence and development of cancer. Studies have shown that oxidative stress, as the converging point of these stimuli, is involved in the mechanical regulation of autophagy process. The regulation of ROS on autophagy can be roughly divided into indirect and direct methods. The indirect regulation of autophagy by ROS includes post-transcriptional and transcriptional modulation. ROS-mediated post-transcriptional regulation of autophagy includes the post-translational modifications and protein interactions of AMPK, Beclin 1, PI3K and other molecules, while transcriptional regulation mainly focuses on p62/Keap1/Nrf2 pathway. Notably, ROS can directly oxidize key autophagy proteins, such as ATG4 and p62, leading to the inhibition of autophagy pathway. In this review, we will elaborate the molecular mechanisms of redox regulation of autophagy in cancer, and discuss ROS- and autophagy-based therapeutic strategies for cancer treatment.

## 1. Introduction

Redox (reduction and oxidation) reaction refers to a type of reaction in which electron transfer occurs. During the cellular redox process, the most important active molecules produced are reactive oxygen species (ROS) and reactive nitrogen species (RNS) [1]. Reactive oxygen species (ROS), as byproduct of aerobic metabolism, are highly active ions and molecules derived from molecular oxygen (O_2_), which include the superoxide anion (O_2_^–^), hydrogen peroxide (H_2_O_2_) and hydroxyl radicals (•OH) [2]. While RNS consist of nitric oxide (NO), nitrogen dioxide radical (NO_2_), nitrite (NO^2−^), nitrate (NO^3−^) and peroxynitrite (OONO^−^) [3,4]. Both ROS and RNS are potent oxidants that can cause intracellular oxidative stress. Initially, elevated ROS levels were thought to be harmful to cells, causing damage to intracellular components and even cell death [5]. However, in-depth studies have shown that the role of ROS in cells is complex and contradictory under both physiological and pathological conditions. ROS maintain redox homeostasis in cells and participate in the regulation of multiple signaling pathways [1]. ROS are associated with cancer, diabetes, cardiopulmonary diseases and many other diseases.

Autophagy is a cellular lysosomal-dependent degradation mechanism that eliminates damaged organelles, misfolded proteins and pathogens to maintain cellular homeostasis [6]. In the face of various stress conditions such as oxidative stress, nutrition restriction, and growth factor deficiency [7], a double-membraned autophagosome encapsulates the cytoplasmic components and delivers them to the lysosomes for degradation and recycling. This degradation and recovery mechanism of autophagy will protect cells from the accumulation of damaged proteins, prevent the attack of microorganisms, and maintain the nutritional supply [8]. Therefore, a basal low level of autophagy is essential for cell survival and homeostasis [9]. A growing number of studies have shown that autophagy is associated with the pathogenesis of various diseases, including cancer. Regulating the activity of autophagy may impact cancer progression, and targeting autophagy in cancer cells has gradually proved to be an effective strategy for cancer treatment [10].

Various cancer cells often exhibit elevated intracellular ROS levels, mainly due to dysregulated metabolism, mitochondrial dysfunction, and oncogenic mutations, etc. [11]. Studies have shown that ROS accumulation plays a variety of roles in cancer. Emerging reports have also confirmed that ROS can directly oxidize and modify autophagy-related proteins, thus regulating autophagy process. In this review, we will summarize the role of ROS as signaling molecules in regulating autophagy process in the survival or death of cancer cells in different settings. We then discuss the potential of molecules or pathways involved in the redox regulation of autophagy as future therapeutic targets for cancer treatment.

## 2. ROS and Cancer

The prominent sources of O_2_^–^ are mitochondrial respiration and membrane-bound NADPH oxidase (NOX) complex [12]. Electron leakage from the mitochondrial electron transport chain (ETC) leads to one-electron reduction of O_2_ and subsequent generation of O_2_^–^ [13]. NOXs are membrane location proteins that catalyze the transfer of electrons from NADPH to O_2_ to form O_2_^–^ [14]. Under the catalysis of superoxide dismutases (SODs), O_2_^–^ can be quickly converted into H_2_O_2_, H_2_O_2_ can then be catalyzed by metal catalyst through the Fenton chemical reaction to generate hydroxyl radicals (•OH) [15]. Beyond that, the accumulation of ROS may lead to the production of RNS [16]. RNS molecules, which are primarily derived from nitric oxide reaction, also participate in various cellular signaling pathways to regulate biological events [17].

**Figure 1:**
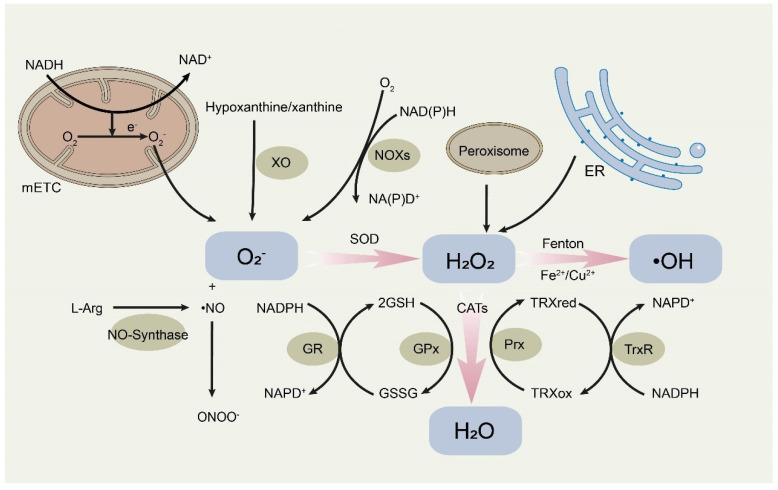
The sources and conversion of ROS. The main sources of ROS include mitochondrial electron transport chain (mETC), NOX complex, peroxisome and endoplasmic reticulum (ER). In order to prevent the damage of ROS, cells are equipped with antioxidant defense system, in which superoxide dismutases (SODs), catalases (CATs), glutathione peroxidases (GPXs), peroxiredoxins (PRXs), and thioredoxins (Trxs) are utilized to maintain the redox homeostasis of cells. NOXs, membrane-bound NADPH oxidases. XO, xanthine oxidase. GSH, glutathione. GSSG, glutathione disulfide. GR, glutathione reductase. TRXred, reduced thioredoxin. TRXox, oxidized thioredoxin.

To counteract the damage of ROS accumulation, cells are equipped with an elaborate antioxidant defense system [18]. The antioxidant defense system mainly comprises nonenzymatic and enzymatic antioxidants to cope with oxidative stress. In general, nonenzymatic antioxidants are usually refer to glutathione (GSH), flavonoids, vitamin A and other small molecules [19]. Among them, GSH is one of the most important antioxidant molecules. After receiving the single electron of ROS or RNS, two GSH molecules generate oxidized dimer (GSSG). GSSG can be regenerated into GSH under the catalysis of glutathione reductase (GR) to maintain intracellular redox homeostasis [20]. Cells also have enzymatic antioxidants, including SODs, catalases (CATs), glutathione peroxidases (GPXs), peroxiredoxins (PRXs), thioredoxins (Trxs) and paraoxonase-2 (PON2), that participate in cytoprotective and detoxification processes [21]. As mentioned above, SODs convert O_2_^–^ to H_2_O_2_, which in turn is catalyzed to produce water by CATs with the participation of GPXs and PRXs [22]. PON2 reduces the release of O_2_^–^ from the inner mitochondrial membrane, thus protecting cells from oxidative stress [23,24]. Studies have proved that PON2 is overexpressed in some solid tumors, and it has been proposed as a therapeutic target for several malignant tumors such as glioblastoma multiforme, bladder cancer and melanoma [25,26,27,28]. The source and conversion of active oxygen are shown in Figure 1.

Intracellular redox state is related to many pathological states, among which cancer is a hot research field. Previous studies observed that the accumulation of ROS can exhibit a variety of contradictory biological effects and participate in the process of cell growth, cell death and metastasis of cancer cells. The specific outcome depends on the genetic background of tumor cells, as well as the distributions, concentrations, and durations of ROS. For example, ROS can promote rapid tumor reproduction by directly oxidizing the cysteine residues of metabolic enzymes involved in glycolysis, fatty acid metabolism and energy homeostasis of cancer cells [29]. In addition, ROS can act as the second messenger in cancer cells, leading to the activation of several carcinogenic pathways or inactivation of cancer inhibition pathways [30]. For example, ROS promote the survival of tumor cells by activating mitogen activated-protein kinase (MAPK)/extracellular-regulated kinase 1/2 (ERK1/2) [31], phosphoinositide-3-kinase (PI3K)/Akt [32] and nuclear factor-κB (NF-κB) pathways [33], inactivating the tumor-suppressor gene p53 [34] and stabilizing the transcriptional factor Nrf2 [35]. ROS can also affect the tumor microenvironment (TME). A number of studies have shown that ROS could promote the conversion of normal fibroblasts into cancer-associated fibroblasts (CAFs) in tumors to augment tumorigenesis. Moreover, ROS could inhibit the function of tumor-infiltrating T-cells to form an immunosuppressive microenvironment [36]. In addition to these tumor-promoting roles, the excessive production of ROS has toxic effects on tumor cells, such as inducing cell cycle arrest, DNA damage, apoptosis and aging [19,37,38]. In addition, ROS are also involved in the regulation of tumor metastasis, which is also contradictory and complex. It has been reported that targeted removal of superoxide by mitoTEMPO (a specific scavenger of mitochondrial superoxide) effectively blocked the lung metastasis of breast cancers in mice [39]. However, studies have also shown that the antioxidant NAPDH-generating enzymes promoted the distant metastasis of human melanoma cells [40].

## 3. Autophagy and Cancer

**Figure 2:**
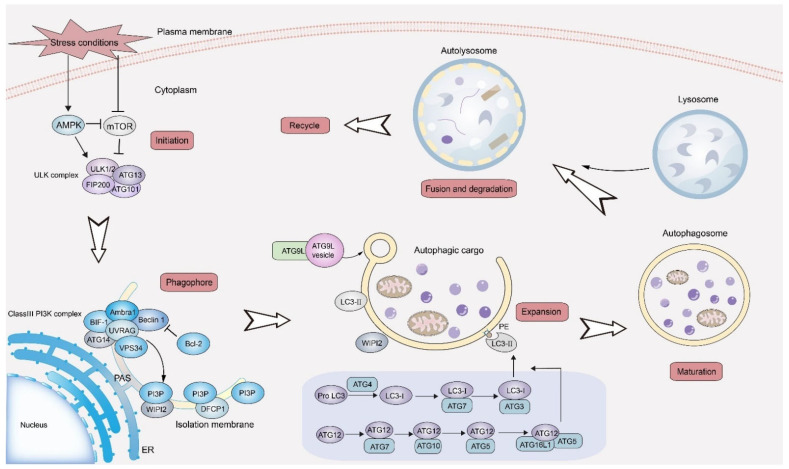
The process of autophagy. Autophagy is initiated in cells by complex regulatory mechanisms under various stress conditions. Through the joint action of multiple protein complexes, the isolation membrane wraps the cargoes, and gradually extends and closes to form autophagosomes. After maturation, autophagosomes fuse with lysosomes to form autolysosomes to degrade the cargoes, realizing the renewal of materials and maintenance of intracellular homeostasis. ER, endoplasmic reticulum. PAS, phagophore assembly site.

The autophagy process firstly originates from the formation of isolation membranes (IM), followed by the recruitment of the core proteins of autophagy (ATGs) to phagophore assembly site (PAS) for assembly [41,42]. Unc-51 like autophagy activating kinase 1 (ULK1) complex, which is activated by inactivation of the mammalian target of rapamycin complex 1 (mTORC1), regulates the composition and activity of class Ⅲ PI3K complex by phosphorylating its components, Beclin 1 and autophagy/beclin-1 regulator 1 (Ambra1) [43]. The activation of the class Ⅲ PI3K complex leads to sequential steps, including recruitment of ATG proteins to PAS for membrane bending, phosphorylation of the lipid head group of phosphatidylinositol to produce phosphatydilinositol-3-phosphate (PI(3)P), and guidance of autophagosome maturation [44]. The elongation of autophagosomes requires two ubiquitin-like conjugation systems: the ATG12-ATG5 and the LC3/ATG8-phosphatidylethanolamine (PE) system. In the first system, as a ubiquitin-like protein, ATG12 firstly conjugates with ATG5 under the action of ATG7 (E1-like enzyme) and ATG10 (E2-like enzyme) [45]. The ATG12-ATG5 conjugate then combines with ATG16L1 to form ATG12–ATG5- ATG16L1 complex, which serves as an E3-like enzyme to couple LC3/ATG8 to PE [46]. Concomitantly, under sequential action of the protease ATG4, the E1-like enzyme ATG7, E2-like enzyme ATG3, and E3-like ligase ATG12-ATG5-ATG16L1 complex, the cytosolic LC3 (LC3-I) is converted to LC3-PE conjugate (LC3-II) [47,48]. Additionally, autophagosome-associated LC3 proteins remain on the membrane of autophagosomes until their fusion with lysosomes [49]. After combining with PE, LC3/ATG8 can recruit a variety of proteins through direct interactions to promote the supplement and transportation of cargoes, as well as lysosomal fusion [50]. Once the membrane of autophagosomes has been sealed, the autophagosomes will undergo a maturation process, during which the autophagosomes are delivered to and fused with the lysosomes [51,52]. Finally, under the action of lysosomal hydrolase and acidic environment, the intracellular contents of autophagosomes are degraded, while the membrane components of autophagosomes are circulated by autophagic lysosome reform (ALR) for cell reuse, so as to realize the metabolic needs and maintenance of intracellular homeostasis [53]. The process of autophagy is also shown in detail in Figure 2.

Autophagy can act as either tumor suppressor or tumor promoter according to the stage of cancer. There are two main types of evidence for autophagy as tumor suppressor. On the one hand, the absence of certain autophagy genes can lead to tumorigenesis. For example, the autophagy gene Beclin 1 (ATG6) exhibits mono-allelic deficiency in 40–75% of sporadic human breast cancers and ovarian cancers, indicating that Beclin 1 is a tumor-suppressor gene [54]. On the other hand, activation of certain oncogenes or inactivation of tumor-suppressor genes will inhibit autophagy. For instance, oncogenic proteins Bcl-2 and Bcl-XL can inhibit autophagy by interacting with Beclin 1 [55]. In addition, overexpression of AKT in a variety of tumors can activate mTOR, leading to blockage of autophagy process. Thus, the combination of pro-autophagic drugs with inhibitors of mTOR, PI3K, or AKT, shows potent anticancer effect [56]. In contrast to its role in constraining tumor initiation, a plethora of studies have shown that autophagy promotes tumor cell survival in advanced cancer. Under the condition of metabolic stress, autophagy can provide energy and essential building blocks for rapidly proliferating tumor cells by circulating intracellular substances, enabling them to thrive in austere microenvironment [57,58]. It has also been shown that autophagy is associated with drug resistance in numerous types of tumor cells. Some residual or metastatic tumor cells can tolerate cytotoxic stress through activating autophagy to become resistance to anticancer drugs [59]. Therefore, therapeutic schemes targeting autophagy inhibition, such as knockdown of LC3 in drug-resistant cells, can sensitize tumor cells to anticancer drugs [60]. Taken together, the autophagy-related pathways are promising targets for cancer treatment, and it is important to fully and deeply understand the complex role of autophagy in cancer.

## 4. Indirect Redox Regulation of Autophagy in Cancer

**Figure 3:**
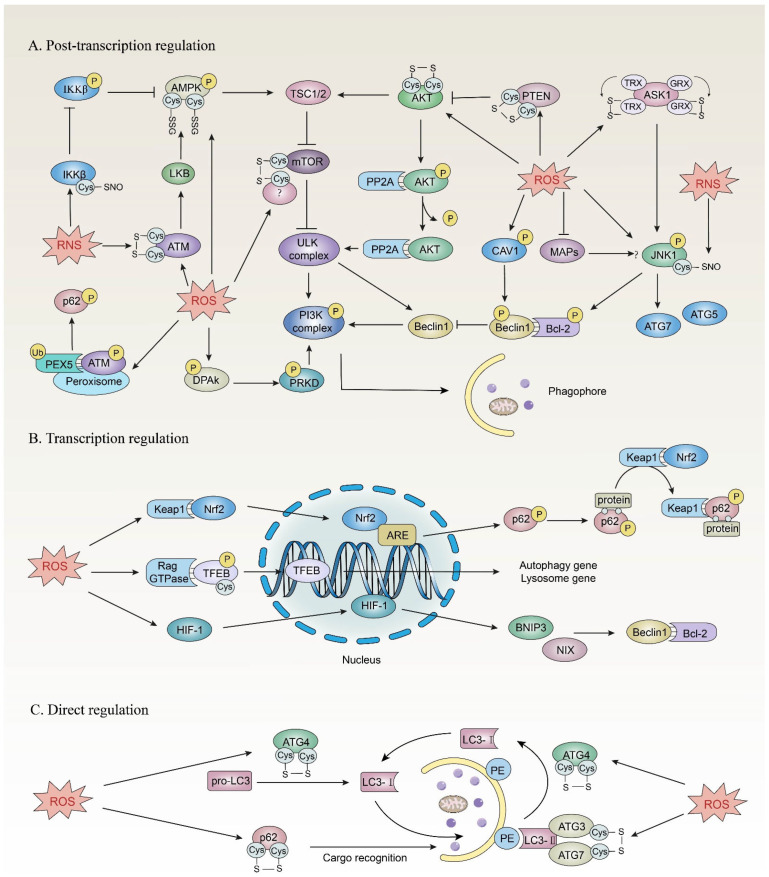
The redox regulation of autophagy. (**A**) mTOR is a core protein countering autophagy initiation. ROS can oxidize and modify AMPK, PTEN, AKT, ATM and other molecules to regulate their activities, and transmit the signal through mTOR to affect autophagy initiation. Beclin 1 usually binds to Bcl-2 to inhibition autophagy initiation, while the oxidative modification of upstream regulatory factors such as JNK1, ASK1 and CAV1 can destroy this interaction to stimulate autophagy. (**B**) In addition, ROS have been demonstrated to affect the autophagy process by regulating the activities and interactions of related transcription factors, including Nrf2, TFEB and HIF-1, resulting in the increased transcription of autophagy-related genes. (**C**) Notably, there is a few evidence for the direct regulation of autophagy by ROS. Currently, it is known that ROS can directly oxidize and modify ATG4 and p62 to participate in the autophagy expansion and cargo recognition processes, respectively.

As mentioned above, both ROS and autophagy are involved in the progression and treatment response of cancer. How ROS regulate autophagy in cancer cells has also become a hot research topic. ROS can indirectly or directly regulate autophagy and thus participate in the development of cancer (Figure 3). Accumulating evidence indicates that reactive cysteines in proteins are molecular switches for transduction of redox signals. The active thiol side chain on cysteine residues of a target protein can be oxidized by ROS to form a sulfenic acid (SOH), which may be further oxidized to form an intramolecular disulfide bond or a S-glutathionylated (SSG) intermediate with glutathione (GSH) [61,62]. These oxidative modifications are reversible, and the oxidation products can be reduced to thiol through the Trx/Trx reductase (or Grx/Grx reductase) with NADPH providing reducing equivalents [63]. Oxidative modifications of reactive cysteines can cause conformational changes of target proteins to transduce redox signals by affecting enzyme activity, protein interaction and further posttranslational modification [29].

### 4.1. Post-Transcriptional Regulation

#### 4.1.1. mTOR Pathway

mTOR is considered to be a core protein in the regulation of autophagy. A classic pathway involved is AMPK/TSC1/2/mTOR signaling axis. In the stress conditions, the increased [AMP]/[ATP] ratio leads to the activation of AMP-activated protein kinase (AMPK), which phosphorylates and activates TSC1/2 protein complex, and sequentially inactivates mTOR [64]. mTOR complex has two different forms—mTORC1 and mTORC2 [65]. mTORC1 is a main regulator of autophagy, and its activation inhibits the initiation of autophagy by phosphorylating ULK1/2 (Ser637 and Ser757) and ATG13 (Ser258) proteins [66]. In addition, PI3K/AKT signaling is also involved in autophagy initiation through regulating mTOR. Extracellular signals such as cytokines and growth factors can be transduced to class I phosphatidylinositol 3-kinase complex (PI3K) through G protein-coupled receptors (GPCRs) and tyrosine kinases (RTKs), and the activated PI3K catalyzes the production of phophatidylinositol-3,4,5-triphosphate (PIP3), thus stimulating AKT and other downstream signaling molecules. Activated AKT promotes the activity of mTORC1 by inhibiting TSC1/TSC2, thereby inhibiting autophagy stimulation [67,68]. Phosphatase and tensin homolog deleted on chromosome 10 (PTEN) can antagonize PI3K signal by dephosphorylating PIP3 [69]. It has been reported that the components of the upstream and downstream signaling of mTOR pathway are often changed to promote tumor progression in various types of cancers, such as amplification of PI3K, loss of PTEN function and overexpression of AKT [70].

The AMPK can act as an energy sensor to monitor the intracellular energy level [71]. Studies have shown that increased levels of intracellular ROS can activate AMPK for the maintenance of redox homeostasis. For example, Wu et al. found that in response to H_2_O_2_ treatment, AMPK was phosphorylated and activated to induce autophagy to counteract oxidative stress, thus enabling cell survival [72,73]. The activation of AMPK by H_2_O_2_ might be due to direct S-glutathionylation of Cys299 and Cys304 [74]. In tumor cells, activated AMPK is thought to maintain redox homeostasis, at least in part, by regulating the initiation of autophagy, thus being closely related to tumor progression [75].

AKT was originally found as an oncogene involved in the regulation of the survival, proliferation, and death pathways of tumor cells [76]. PTEN, a phosphatase that opposes forward PI3K signaling, can positively regulate autophagy and exert anti-tumor effect by inhibiting AKT activation [77]. Leslie et al. demonstrated that H_2_O_2_ could oxidize and deactivate PTEN in glioblastoma cells. Inactivation of PTEN caused an increased intracellular PIP3 level, which in turn led to the activation of the downstream AKT [78]. Further study showed that Cys124 of PTEN specifically formed disulfide bond with Cys71 in response to H_2_O_2_ treatment [79]. In addition, some studies showed that ROS could directly regulate the activity of AKT. Under oxidative stress, AKT formed intramolecular disulfide bonds between Cys297 and Cys311, leading to its dephosphorylation and inactivation by binding to protein phosphatase PP2A [80]. However, whether AKT-regulated autophagy promotes tumor survival by maintaining redox homeostasis or plays a cytotoxic role by inducing tumor cell death remains to be explored.

Walker’s group reported that ROS could oxidize and activate ataxia-telangiectasia mutated (ATM) to repress the downstream TSC2-mTOR signaling pathway, thus initiating autophagy [81]. Different from the classical pathway for ATM activation via DNA double-strand breaks in the nucleus, it has been reported that H_2_O_2_ treatment could directly oxidize ATM through forming a disulfide-cross-linked dimer at Cys2991, leading to ATM activation [82]. It has been reported that ATM could induce intestinal cell death of Caenorhabditis elegans by stimulating autophagy in response to oxidative stress [83]. Walker’s group later demonstrated that ROS may also be involved in the cargo identification of autophagy [84]. The peroxisome import receptor PEX5 could bind and re-localize ATM to the peroxisomes. ROS-induced ATM activation promotes the mono-ubiquitination of Lys209 by phosphorylating the Ser141 site of PEX5. The autophagy adapter p62 then recognized the ubiquitinated PEX5 to direct the autophagosomes to engulf the peroxisomes [85].

In addition to ROS, it has been demonstrated that nitric oxide (NO) could S-nitrosylate IKKβ and reduce its phosphorylation, thus preventing AMPK phosphorylation and impairing autophagy initiation [86]. Interestingly, different from the traditional viewpoint of RNS as an autophagy inhibitor, some studies have pointed out that NO could induce autophagy. In breast cancer cells, nitrogen stress caused rapid activation of the ATM damage-response pathway and downstream LKB1, which ultimately inhibited AMPK/TSC1/2/mTOR pathway to induce autophagy [87]. Therefore, cancer cells are particularly sensitive to nitrogen stress, and nitrosative stress-induced autophagy might be a promising therapeutic target for cancer treatment.

The redox regulation of the mTOR signaling pathway is not limited to the upstream molecules. Recent studies have shown that ROS could directly oxidize mTOR. Oka et al. found that an intermolecular disulfide bond formed at Cys1483 of mTOR in cultured cardiomyocytes following H_2_O_2_ treatment, resulting in the mTOR inactivation and the inhibition of downstream signaling [88]. Although the oxidation of mTOR has not been reported in tumor cells, given the important role of ROS, mTOR signaling and autophagy in tumor progression, it is reasonable to presume that ROS might be involved in tumor progression by directly oxidizing mTOR to regulate autophagy.

In conclusion, these observations suggest that the mTOR signaling pathway is a key regulatory step in ROS-induced autophagy, but the exact molecular mechanism between ROS-induced autophagy and mTOR signaling in cancer remains to be further investigated.

#### 4.1.2. Beclin 1

Beclin 1 is an indispensable protein in the process of autophagy, and Beclin 1 gene is also the first discovered gene to link autophagy with human tumors [89]. The Bcl-2 family of proteins, Bcl-2, Bcl-xL and Mcl-1, which are anti-apoptotic proteins, can bind to the Bcl-2 homology 3 (BH3) domain of Beclin 1 to exert a rheostat effect for autophagy [90]. Under normal conditions, Beclin 1 binds to Bcl-2. When separating from Bcl-2, Beclin 1 can form PI3KC3 complex with Vps15, Vps34 (also called type III PI3K) and other proteins like Ambra1, to regulate the initiation of autophagy [42]. Studies have shown the downregulation of Beclin 1 and upregulation of Bcl-2 in many types of cancers, both were closely related to poor prognosis [91,92]. It has been reported that Bcl-2 antagonized the autophagy pathway, subverted the internal protein quality and genome stability and promoted the growth of breast cancer cells, indicating that Beclin 1 and Bcl-2 can be served as attractive targets for cancer therapy [93].

As mentioned above, the dissociation of Bcl-2/Beclin 1 complex is essential for the initiation of autophagy, which is regulated by key kinases. For example, nutritional stress-activated c-Jun N-terminal protein kinase 1 (JNK1, also known as mitogen-activated protein kinase 8, MAPK8), promoted multisite phosphorylation of Bcl-2, leading to the dissociation of Bcl-2 from Beclin 1, thereby inducing autophagy [94]. The typical MAPK pathway consists of three sequentially activated MAPK family members, including MAPK kinase kinase (MAPKKK), MAPK kinase (MAPKK) and MAPK [95]. ROS are considered as a potent inducer for the activation of the MAPK family members. It has been reported that ROS inactivated MAPK phosphatase 3 (MKP-3) by oxidizing Cys293 to sulfenic acid, and then continuously activated JNK1 [96]. Further studies have shown that ROS-mediated JNK activation could induce autophagy by upregulating ATG5 and ATG7, which is crucial for oncogenic K-Ras-induced malignant cell transformation [97]. Inhibition of oxidative stress with antioxidants, or ATG5 or ATG7 knockdown using shRNA obviously inhibited autophagy and prevent malignant transformation [98]. ROS could also activate JNK1 by oxidizing ASK1 (a ubiquitously expressed MAPKKK). Thioredoxin is an internal inhibitor of ASK1, and its redox state determines the activity of ASK1. ROS can cause thioredoxin to form an intramolecular disulfide between Cys32 and Cys35 residues, leading to the dissociation of oxidized thioredoxin from ASK1 and the subsequent activation of ASK1 [99,100]. Similarly, researchers also found that ROS could oxidize glutaredoxin, thus promoting its dissociation from ASK1 and causing JNK1 activation [101]. It has been reported that endogenous nitric oxide could cause S-nitrosylation at Cys116 of JNK1, which is proved to be a critical cysteine residue for the thiol-redox regulation of JNK1, resulting in JNK1 inactivation [102]. Through inhibiting JNK1-mediated Bcl-2 phosphorylation, NO increases the interaction of Bcl-2 with Beclin 1 to inhibit autophagy initiation [86].

Some other regulatory factors have also been reported to be sensitive to redox state and regulate Beclin 1-mediated autophagy. For example, H_2_O_2_ treatment promoted the phosphorylation of CAV1 (Caveolin-1, an integral membrane protein) at Tyr14. The phosphorylated CAV1 then interacted with Beclin 1-Vps34 complex through its scaffolding domain to promote the formation of autophagosomes. When the phosphorylation of CAV1 was reduced by PTEN1, or CAV1 was knocked out, autophagy flux was decreased [103]. However, in contradiction with above report, it has been proved that ROS could inhibit autophagy initiation through TRPM2-Ca^2+^-CAMK2-Beclin 1 cascade. In detail, ROS triggered TRPM2-dependent Ca^2+^ influx, mediated the oxidation of two adjacent methionine residues (Met281 and Met282) in CAMK2, and then phosphorylated Beclin 1 at Ser295. The phosphorylation of Beclin 1 enhanced its interaction with Bcl-2, leading to the inhibition of autophagy at early stage [104].

Notably, the regulation of autophagy by ROS can also be achieved by regulating Vps34 and its interaction with Beclin 1. Eisenberg-Lerner et al. demonstrated that under oxidative stress, PKD was phosphorylated and consequently activated by DAPK (death-associated protein kinase) [105]. Activated PKD then phosphorylated and activated Vps34 leading to the production of PI3Pand formation of autophagosome. Another example is cannabidiol, an antineoplastic agent, promoted ROS generation and induced cell death of breast cancer cells. In addition, cannabidiol promoted the dissociation of Beclin 1 and Bcl-2, and enhanced the interaction of Beclin 1 with Vps34 to induce autophagy. Scavenging of ROS led to the blockage of cannabidiol-induced autophagy, suggesting that cannabidiol regulate autophagy through inducing oxidative stress [106]. Moreover, cannabidiol-induced autophagy did not maintain homeostasis in breast cancer cells, but rather promoted cell death and inhibited tumor development, which once again suggest the double-edged sword effect of autophagy in tumor cells.

### 4.2. Transcriptional Regulation

#### 4.2.1. p62/Keap1/Nrf2

In addition to the effects of ROS on the oxidative modification and interaction of the upstream regulatory molecules in the autophagy process, ROS can also regulate autophagy by affecting gene transcription. Nrf2, a key transcription factor in the antioxidant defense system, activates the transcription of SOD, catalase, GR, heme oxygenase (HO-1) and other antioxidant enzymes to protect cells from oxidative stress [107]. Under unstressed conditions, Nrf2 interacts with the Kelch-like ECH-associated protein 1 (Keap1), which mediates Nrf2 ubiquitination and promotes the proteasomal degradation of Nrf2 [108]. In response to oxidative stress, Nrf2 dissociates from Keap1 and subsequently enters the nucleus [35]. Nuclear Nrf2 specifically binds to the antioxidant response element (ARE) located in the promoter of the autophagy adaptor sequestosome 1 (p62/SQSTM1), and then promotes the transcription of p62 [109]. Phosphorylation of p62 increases the binding affinity for Keap1 and competitively separates Nrf2 from Keap1, thereby forming a positive feedback loop [110,111].

Elevated p62 levels are essential for tumorigenesis. Mathew et al. found that autophagy-defective cells resulted in the accumulation of p62 and promoted tumorigenesis [112]. The research on autophagy-defective mouse models also provided strong evidence for this viewpoint. For example, accumulation of p62 and Keap1 protein aggregates and sustained activation of Nrf2 were observed in liver-specific autophagy-deficient mice and demonstrated to be associated with the hepatocarcinogenesis [113]. In addition to tumorigenesis, the upregulation of p62 was also associated with cancer progression and therapeutic resistance [114]. It should be mentioned that a variety of natural and synthetic compounds, such as γ-tocopherol (γ-TmT) and sulforaphane (SFN), could elevate the expression of Nrf2 to protect cells from oxidative stress, thereby significantly reducing the incidence of prostate cancer in transgenic adenocarcinoma of mouse prostate (TRAMP) mice [115]. Taken together, drugs targeting the p62/Keap1/Nrf2 signaling pathway, such as p62 inhibitors and Nrf2 modulators have potential to become new strategies for tumor treatment.

#### 4.2.2. TFEB

Transcription factor EB (TFEB) is an important transcription factor involved in lysosomal biogenesis and autophagy [116]. TFEB can interact with Rag GTPases and be recruited to lysosomes in an amino acid-dependent manner [117]. Later, TFEB is phosphorylated by mTOR at Ser211 and sequestered in the cytosol through binding with 14-3-3 proteins [85]. Under stress, inactivation of mTOR results in dephosphorylation and nuclear translocation of TFEB, leading to the transcription of genes required for lysosomal biogenesis and autophagy induction [118].

Recently, Wang et al. revealed a new mechanism that TFEB can be directly oxidized and activated by ROS, thus activating autophagy [119]. They found ROS directly oxidized TFEB at Cys212, thus abolishing the interaction between Rag GTPases and TFEB and inducing rapid nuclear translocation of TFEB independent of mTOR inhibition. It was found that H_2_O_2_ treatment increased the expression of genes involved in autophagy-lysosome system, indicating that the activation of TFEB was required for ROS-driven autophagy. Dysfunction in autophagy-lysosome system is closely associated with many human diseases, and therapeutic strategies targeting this process have also been developed. For example, salidroside, an active ingredient separated from the traditional Chinese herbal medicine Rhodiola rosea, promoted the nuclear translocation of TFEB by inducing ROS accumulation, leading to the induction of autophagy and apoptosis in human chondrosarcoma cells [120].

#### 4.2.3. HIF-1

Hypoxia is often present in the tumor area due to excessive proliferation, high oxygen consumption, and limited extent of tumor angiogenesis [121]. Hypoxia inducible factor-1 (HIF-1) is a key transcription factor for cancer cells to adapt to the hypoxic environment, and has been proven to be an important cancer drug target [122]. This transcription factor has oxygen-dependent instability, which degrades rapidly under normoxic conditions, but can exist stably under anoxic conditions or generation of mitochondrial ROS [123]. HIF-1 can drive the transcription of hundreds of genes involved in angiogenesis, glucose metabolism, migration and invasion in tumor cells [124]. Kobayashi et al. demonstrated that prolonged hypoxia increased the levels of ROS, inducing the expression of redox factor-1 (Ref-1) to activate HIF-1 [125]. Some studies suggest that the mechanisms for the occurrence of hypoxia in tumor is that the production of ROS can cause vascular endothelial damage [126]. Hypoxia could rapidly induce the survival response of autophagy through HIF-1. In detail, HIF-1 induced the transcription of pro-apoptotic genes BNIP3 and NIX to compete for Beclin 1 binding with Bcl-2, thus releasing Beclin 1 to induce autophagy [127]. Of interest, NO has been reported to promote the stabilization and transcriptional activity of HIF-1 by S-nitrosation of Cys800 [128]. Given the multiple roles of HIF-1 in autophagy and tumor progression, inhibitors of HIF-1 and its downstream pathways have also become promising targets for cancer treatment.

## 5. Direct Redox Regulation of Autophagy in Cancer

### 5.1. ATG4

Although there is a large amount of data supporting the viewpoint of redox regulation of autophagy, the evidence for direct regulation of autophagy-related proteins by redox signaling remains limited. ATG4 is the first ATG family protein identified to be directly oxidized by ROS. There are four different ATG4 homologs expressed in human: ATG4A, ATG4B, ATG4C and ATG4D, among which ATG4B is crucial for the autophagic process [129]. ATG4 plays a key role in the regulation of the ATG8/ LC3 lipid conjugation system. First, the cysteine protease ATG4 cleaves pro-LC3 to expose a glycine residue (Gly120) near the C-terminus to form conjugates with PE by ubiquitin-like systems [130]. Second, after autophagosome closure, membrane-localized LC3-II is delipidated by ATG4 at the bond between Gly120 residue and PE to recycle LC3-I [131].

Given the dual role of ATG4, its activity should be tightly regulated. To investigate this regulatory mechanism, Scherz-Shouval and colleagues found that recombinant HsAtg4A was active only in the presence of the reductant DTT, suggesting that the activity of ATG4 might be regulated by redox potential [132]. The authors went on to find that ROS, specifically H_2_O_2,_ could directly oxidize and regulate ATG4 in nutrient starvation-induced autophagy [133]. They specified Cys81 of ATG4A and Cys78 of ATG4B as critical sites for this regulation. Mutation of these two sites to serine significantly prevented LC3 lipidation and autophagosome formation. Taken together, starvation-induced oxidative signals caused inactivation of ATG4 at the site of autophagosome formation, thus promoting lipidation of ATG8 to facilitate autophagosome formation. However, since Cys81 is not conserved, the molecular mechanism is distinct in different species. It has been reported that redox regulation of ATG4 in yeast cells was mediated by the formation of a single disulfide bond between Cys338 and Cys394 [134]. Similarly, Li et al. recently found that the mechanism of ATG4B inactivation upon exposure to oxidants in HEK293 and Hela cells was the formation of intermolecular disulfide bond between Cys292 and Cys361 [135]. Mutation of both Cys292 and Cys361 reduced the redox sensitivity of ATG4B and increased autophagic flux. In addition to ROS, it has been observed that high glucose level could induce RNS accumulation, which mediated the impaired synthesis of autophagosomes to inhibit autophagic flux. The activity of ATG4B was compromised by RNA-mediated S-nitrosation at Cys189 and Cys292. The impaired autophagy mediated by ATG4B S-nitrosation led to neurotoxicity in response to high glucose level [136].

Interestingly, Frudd et al. reported a different thiol-dependent process for the negative regulation of autophagy [137]. They showed that H_2_O_2_ treatment directly oxidized ATG3 (Cys264) and ATG7 (Cys572) instead of ATG4 to prevent LC3 lipidation. The discordance in these studies might be attributable to the difference of autophagy-inducing systems and ROS levels [85]. It was suggested that ATG3 and ATG7 might have higher redox sensitivity than ATG4. In detail, at low level of H_2_O_2_, ROS serve as signaling molecules to inhibit autophagy induction. When ROS reach higher levels, ATG4 oxidation becomes dominant, and promotes autophagy induction to prevent oxidative damage. However, this speculation needs further investigation.

Increasing evidence shows that the expression of ATG4 is aberrant in various types of tumors, suggesting it is a potential anticancer target. For example, exposure to cadmium (Cd) significantly increased ROS production, elevated ATG4 expression, activated autophagy and promoted cell growth, migration and invasion in human lung glandular cancer [138]. Another agonist of ATG4, flubendazole, has also been found to induce autophagic cell death and ROS production, leading to the inhibition of breast cancer cell growth [139]. Notably, a drug repurposing screening showed that tioconazole is an inhibitor of ATG4, which could stably occupy the active site of ATG4 in its open form to reduce the autophagic flux of cancer cells. Moreover, tioconazole suppressed tumor growth and sensitize tumor cells to starvation and chemotherapeutic drugs [140]. Similar findings were also observed in another ATG4 antagonist, the NSC185058, which negatively affected the development of osteosarcoma by inhibiting autophagy [141]. The above data indicate that both agonists and antagonists of ATG4 have tumor-therapeutic potential and once again demonstrate the dual role of autophagy. In the future drug discovery targeting ATG4, it may consider the type and stage of tumor and the specific role of ATG4. In conclusion, targeting ROS-dependent regulation of ATG4 for autophagy is a new approach for tumor treatment, and its specific molecular mechanism and targeting strategy need to be further investigated.

### 5.2. SQSTM1/p62

As mentioned above, ROS can increase the transcription of prototypic autophagy receptor SQSTM1/p62 and thus affect the autophagy process. Recently, it was found that two oxidation-sensitive cysteine residues in p62 could ungergo direct redox modification [142]. In response to the treatment of H_2_O_2_ or PR-619 (a redox cycler known to produce H_2_O_2_), the Cys105 and Cys113 were oxidized to promote the formation of p62 disulphide-linked conjugates (DLC), allowing p62-dependent aggresome formation. Furthermore, p62 oxidation and oligomerization was proved to promote autophagosome biogenesis and degradation of ubiquitylated autophagic cargoes, and activate pro-survival function of autophagy in stress conditions.

However, many issues regarding p62 oxidation and oligomerization remain to be resolved. For example, what is the potential molecular mechanism of DLC affecting the oligomerization of p62? In addition, redox sensitivity of p62 may occur in age-related pathology in humans, including aging, cancer and ischaemia/reperfusion injury. How the oxidation of p62 mediates autophagy and participates in disease processes still requires formal testing in vivo. Compared with p62/Keap1/Nrf2 pathway, whether p62 oxidation plays an antagonistic or synergistic role in cancer progression requires further investigation. Nevertheless, pro-survival autophagy mediated by p62 oxidation may also become a new anticancer target.

## 6. Cancer Therapy

**Figure 4:**
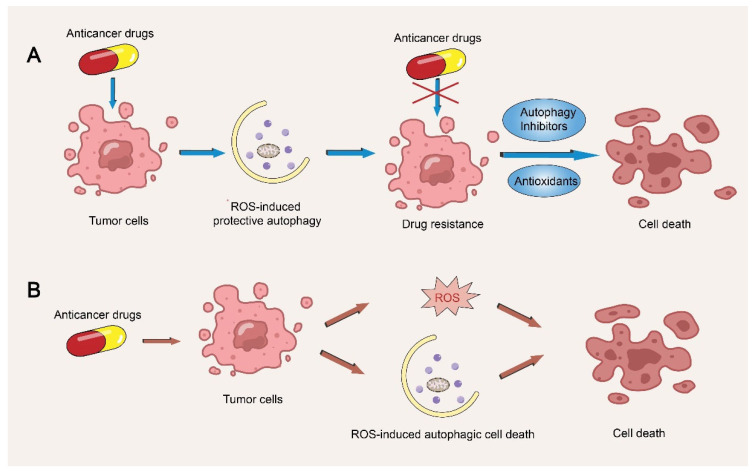
Two anticancer strategies based on the regulation of autophagy by ROS. (**A**) During the treatment with some anticancer drugs, ROS-mediated protective autophagy could be induced in tumor cells, leading to the development of drug resistance. Autophagy inhibitors such as chloroquine (CQ) or antioxidants such as NAC could enhance the sensitivity of tumor cells to anticancer drugs. (**B**) Some anticancer drugs, such as bruceine D and resveratrol, exerted cytotoxicity in cancer cells by promoting ROS accumulation and ROS-induced autophagic cell death.

In view of the contradictory roles of autophagy and ROS in cancer treatment, a series of clinical trials have been conducted. As shown in Figure 4, the first type is based on the observation that ROS induces protective autophagy leading to the drug resistance in tumor cells. The use of inhibitors of autophagy or antioxidants may enhance or restore the cytotoxicity effect of anticancer drugs [143]. To date, most pre-clinical studies and clinical trials support the use of the autophagy inhibitors chloroquine (CQ) or hydroxychloroquine (HCQ) in anticancer therapy, either as a single agent or in combination with other anticancer drugs [144]. One example is ciclopirox olamine (CPX), a potential anticancer agent, that has been demonstrated to induce cell protective autophagy through ROS-mediated JNK activation in human rhabdomyosarcoma (Rh30 and RD) cells. Chloroquine enhanced CPX-induced cell death, suggesting that the combination of autophagy inhibitors with CPX is a novel strategy for the treatment of rhabdomyosarcoma [145]. Similarly, antioxidants may be able to resverse tumor-drug resistance. For example, anticancer drug apogossypolone (ApoG2) could activate the ROS/JNK/ERK signaling pathway to induce protective autophagy in human hepatocellular carcinoma (HCC) cells, and the use of antioxidant (N-acetylcysteine, NAC) increased the sensitivity of HCC cells to ApoG2 [146]. Ginsenoside Rg3, the main active component of ginseng, has been reported to suppress proliferation and induce apoptosis of Lewis lung carcinoma (LLC) cells by reducing ROS levels [147]. When combining with cisplatin, Rg3 could prevent intracellular ROS accumulation induced by cisplatin, enhance the susceptibility of colorectal cancer cells to cisplatin [148]. Some other natural antioxidants such as resveratrol and carotenoid have also been reported to remove endogenously generated radicals and reduce the incidence of cancer [149,150,151,152]. However, in view of the dual roles of ROS in autophagy, the role of antioxidants also varies with genetic, epigenetic and microenvironmental variations [153]. There are some literatures reported that antioxidant supplementation during chemotherapy or radiotherapy reduced the survival time of patients [154]. One possible explanation is that the use of antioxidants weakens the cytotoxicity of antitumor drugs by inhibiting drug-induced ROS accumulation. Therefore, antioxidants as an anticancer strategy should be carefully considered before entering clinical use. In contrast, the second cancer therapy strategy is to exploit ROS and autophagy to achieve cytotoxicity. Ionizing radiation (IR) is one of the common means of cancer treatment, and it can exert cytotoxicity by producing ROS. Ionizing radiation has also been reported to induce autophagy in certain types of cancer cells, such as malignant glioma cells and pancreatic cancer cells [155,156]. In addition to ionizing radiation, some drugs were reported to stimulate ROS production to trigger autophagic cell death. 2-methoxyestradiol (2-ME), a natural metabolite of estradiol, has been identified as a promising anticancer agent [157]. Chen et al. found that 2-ME induced oxidative stress in the transformed cell line HEK293 and cancer cell lines U87 and HeLa, thereby increasing autophagy-induced cell death [158]. Similar data were obtained with bruceine D and resveratrol, both of which could induce apoptosis through ROS-triggered autophagy in lung cancer and colon cancer, respectively [159,160].

Determining the redox regulation of autophagy in tumor cells is an important and challenging endeavor. From the currently available reports, the cellular consequences of ROS-induced autophagy are cell type-specific and treatment-dependent. Therefore, researchers should implement precision or personalized medicine to provide tailored treatment for cancer patients. At present, several key issues need to be further solved, including finding simple and rapid approaches to quantify ROS and autophagy levels in vivo, exploring the exact mechanism of redox regulation of autophagy, and determining the most effective approaches to target ROS-mediated autophagy for cancer therapy [143,161]. Many known redox regulation mechanisms of autophagy still lack direct evidence in tumor cells, especially in tumor tissues. Only when the relationship between ROS, autophagy and cancer is fully understood can it be effectively exploited in pharmaceutical and medical research areas.

## 7. Conclusions and Perspectives

Both ROS and autophagy are thought to play double-edged roles in cancer. ROS act as both signaling and damaging molecules. Autophagy can rescue the cell from toxic stress, but it can cause autophagic cell death under certain conditions. Both ROS and autophagy are dysregulated in tumors, and it is also now widely accepted that ROS is involved in the regulation of autophagy, leading to tumor progression or tumor suppression. Here, we review the complex mechanism underlying redox regulation of autophagy in tumor cells and elucidates potential targets for the treatment of cancer. In conclusion, it is, therefore, now clear that future cancer treatment will move towards precision medicine with an extensive evaluation of the genetic or biochemical background of each patient and tumor category (tissue, stage, autophagy levels, ROS levels). Although many problems and challenges remain, new anticancer approaches targeting ROS-mediated autophagy will be continually developed to provide hope for cancer therapy.
